# Serum BP180 and BP230 autoantibodies in Alzheimer’s disease: diagnostic and cognitive associations in a retrospective study

**DOI:** 10.3389/fnagi.2026.1757497

**Published:** 2026-06-02

**Authors:** Sha Jin, Jie Yu, Huai-Li Liu, Cong Wang, Feng-Ming Zheng

**Affiliations:** Department of Geriatrics, Hangzhou Third People’s Hospital, Hangzhou, China

**Keywords:** Alzheimer’s disease, biomarkers, BP180, BP230, MMSE

## Abstract

**Background:**

To assess the prevalence and diagnostic value of serum anti-BP180 and anti-BP230 antibodies in Alzheimer’s Disease (AD) and their relationship with cognitive function.

**Methods:**

We conducted a retrospective case–control study comprising 279 participants, including patients with clinically diagnosed AD and neurologically healthy controls, who were consecutively enrolled at the Third People’s Hospital, Hangzhou, China, between 2022 and 2024. Serum levels of anti-BP180 and anti-BP230 antibodies were quantified using enzyme-linked immunosorbent assays (ELISAs). Among patients with AD, the relationship between cognitive function, assessed by the Mini-Mental State Examination (MMSE), and serum antibody concentrations was further evaluated.

**Results:**

After adjustment for age and sex, serum BP180 levels were significantly higher in AD patients compared with healthy controls (*β* = 2.257, 95% CI: 1.102–3.412, *p* < 0.001). Similarly, BP230 levels were also significantly elevated in AD patients (*β* = 3.432, 95% CI: 0.395–6.468, *p* = 0.027). Multivariable logistic regression analysis identified BP180 (odds ratio [OR] = 1.149, 95% CI: 1.056–1.275, *p* = 0.004), age (OR = 1.176, 95% CI: 1.110–1.254, *p* < 0.001), and female sex (OR = 5.318, 95% CI: 2.853–10.272, *p* < 0.001) as independent predictors of AD. A predictive model incorporating BP180, age, and sex was subsequently developed, demonstrating good diagnostic performance with an area under the curve (AUC) of 0.798 (95% CI: 0.734–0.862). Compared with BP180 alone (AUC = 0.591) or BP230 alone (AUC = 0.598), the multivariable model showed significantly improved discriminative ability. In addition, both BP180 (*p* < 0.001) and BP230 (*p* = 0.003) levels were significantly and negatively correlated with MMSE scores.

**Conclusion:**

The multivariable model incorporating BP180, age, and sex demonstrated good discriminative performance in distinguishing AD patients from controls and outperformed individual biomarkers. Integrating clinical and serological markers may improve the diagnostic performance for AD.

## Introduction

Alzheimer’s disease (AD) is the most common neurodegenerative disorder worldwide, characterized by progressive cognitive decline, amyloid-*β* (Aβ) deposition, and neurofibrillary tangles composed of hyperphosphorylated tau protein (Scheltens et al., 2021). In recent years, the association between neurodegenerative diseases and autoimmune skin disorders has garnered increasing research attention (Försti et al., 2016; Messingham et al., 2016; Ungprasert et al., 2016; Nieto-Benito and Suárez-Fernández, 2025; Bastuji-Garin et al., 2011; Zhang et al., 2021), with the comorbidity between bullous pemphigoid (BP) and AD emerging as a particular focus of investigation.

BP is an autoimmune blistering disorder predominantly affecting the elderly, characterized by autoantibodies against the hemidesmosomal proteins BP180 (also known as collagen XVII) and BP230 (also known as dystonin-e) (Nishie, 2014). In BP pathogenesis, autoantibodies targeting the NC16A domain of collagen XVII play a central role, whereas the clinical relevance of anti-BP230 antibodies remains less clear. Epidemiological and clinical studies have consistently shown that AD patients have a significantly elevated risk of developing BP (Seppänen, 2013; Julio et al., 2018; Försti et al., 2014; Brick et al., 2014; Kibsgaard et al., 2017). Importantly, both BP180 and BP230 are expressed not only in the cutaneous basement membrane but also in the central nervous system, providing a molecular basis for the link between the two disorders (Seppänen, 2013; Seppänen et al., 2006; Li et al., 2009).

A substantial proportion of AD patients have been found to harbor serum autoantibodies against BP180 and or BP230 (Seppänen, 2013; Kokkonen et al., 2017; Wang et al., 2021). A study by Kokkonen et al. (2017) reported that 18% of AD patients were positive for anti-BP180 autoantibodies, significantly higher than the 3% positivity in controls (*p* = 0.019). Moreover, the levels of anti-BP180-NC16A autoantibodies were inversely correlated with cognitive function scores, suggesting that BP180 autoimmunity may be involved in the neurodegenerative progression of AD. This indicates the potential of circulating anti-BP180 antibodies to serve as peripheral blood biomarkers for AD disease activity. Furthermore, Wang et al. (2021) detected anti-BP180 antibodies in 47.9% of 48 AD patients, and 47.8% of those positive sera recognized a 180-kDa protein in human brain extracts, confirming that BP180 serves as a shared autoantigen in AD and BP. However, unlike in classic BP, the epitopes recognized by AD-derived autoantibodies are often located in intracellular or mid-extracellular regions of BP180 rather than the pathogenic NC16A domain. Moreover, these antibodies generally fail to bind native, conformationally intact BP180 at the cutaneous basement membrane in indirect immunofluorescence assays, which may explain why many AD patients with such autoantibodies do not exhibit cutaneous symptoms (Tuusa et al., 2019). Regarding BP230, case reports have also indicated an association with dementia, including AD and dementia with Lewy bodies (Zheng et al., 2018). A meta-analysis further revealed that the gene encoding BP230, DST, is upregulated in both diabetic and AD models, implying its potential role in a common pathological network linking diabetes, BP, and dementia (Cheng et al., 2019).

To date, systematic studies examining the relationship between AD and autoantibodies against BP180 and BP230 in Chinese populations remain limited. To validate and extend these findings specifically in a Chinese cohort, we conducted a large-scale analysis of 279 samples. To our knowledge, this represents the largest cohort study to date investigating anti-BP180 and anti-BP230 autoantibodies in Chinese patients with Alzheimer’s disease.

## Methods

### Patients

This retrospective study included a total of 279 participants, comprising patients with AD and cognitively normal controls. The clinical diagnosis of AD in this study strictly adhered to the core clinical diagnostic criteria proposed by McKhann (Mckhann et al., 2011) and Albert et al. (2011) in 2011, as outlined by the National Institute on Aging–Alzheimer’s Association (NIA-AA). Clinical and laboratory data were obtained from Third People’s Hospital, Hangzhou, China between January 2022 and December 2024. Only participants with available serological test results for BP180 and BP230 were included, encompassing both AD patients and non-AD individuals. Participants with coexisting BP were excluded. Serum levels of BP180 and BP230 autoantibodies were measured, and cognitive function in AD patients was assessed using the Mini-Mental State Examination (MMSE).

The study protocol was reviewed and approved by the Ethics Committee of Hangzhou Third People’s Hospital (approval number: 2025KA257). Given the retrospective nature of the study and the use of anonymized data, the requirement for written informed consent was waived.

### Enzyme-linked immunosorbent assay (ELISA)

The serum levels of IgG autoantibodies against BP180-NC16A-4X and BP230-CF were quantitatively measured using the respective EUROIMMUN ELISA kits (Anti-BP180-NC16A-4X IgG ELISA and Anti-BP230-CF IgG ELISA) according to the manufacturer’s instructions. A cutoff value of ≥20 RU/mL was applied to define positivity for both autoantibodies.

### Statistical analysis

All statistical analyses were performed using R software (version 4.5.1; R Foundation for Statistical Computing, Vienna, Austria). Continuous variables were presented as medians (ranges) and compared using non-parametric tests, while categorical variables were compared using chi-square tests. Serum BP180 and BP230 autoantibody levels were compared between groups using the Mann–Whitney *U* test. Spearman’s rank correlation was employed to examine associations between continuous antibody levels and cognitive scores.

Covariate balancing propensity score (CBPS)–based inverse probability weighting (IPW) was performed to adjust for potential confounding by age and sex. Balance diagnostics demonstrated excellent covariate balance, with all standardized mean differences (SMDs) below 0.1 after weighting. The effective sample size was reduced in the weighted pseudo-population, indicating appropriate weight distribution. In the weighted pseudo-population, IPW–adjusted survey-weighted linear regression models were applied to assess differences in serum BP180 and BP230 levels between AD patients and controls. Receiver operating characteristic (ROC) curve analysis was performed to evaluate the diagnostic performance of the multivariable model, and the area under the curve (AUC) was calculated with 95% confidence intervals.

Group comparisons based on antibody positivity, defined as antibody levels exceeding the manufacturer-recommended threshold of 20 RU/mL, were conducted using chi-square tests. Two-tailed *p* values ≤0.05 were considered statistically significant.

## Results

The demographic and clinical characteristics of the study population are shown in Table 1. A total of 279 participants were included in this study, comprising 199 patients with AD and 80 cognitively normal controls. The median age of all participants was 89 years. The median age was 85 years (range 65–99) in the control group and 91 years (range 77–99) in the AD group, with a significant difference between the two groups (*p* < 0.001). In the control group, there were 56 males and 24 females, whereas females were the majority in the AD group (n = 127, 63.8%), with 72 males; the sex distribution differed significantly between the two groups (*p* < 0.001).

**Table 1:** Baseline characteristics.

	Characteristics		
	Control group (*N* = 80)	AD patients (*N* = 199)	*p-*value
Age, med (range), years	85 (65–99)	91 (77–99)	<0.001
Sex (M/F)	56 (70.0%)/24 (30.0%)	72 (36.2%)/127 (63.8%)	<0.001
MMSE, med (range)	NA	13 (8–29)	
BP180 (RU/mL), med (range)	3 (2–21)	3 (2–52)	0.014
BP180 −/+ (20 RU/mL)	79 (98.8%)/1 (1.3%)	192 (96.5%)/7 (3.5%)	0.446
BP230 (RU/mL), med (range)	2 (2–48)	6 (2–98)	0.009
BP230 −/+ (20 RU/mL)	71(88.8)/9 (11.3%)	178 (89.4)/21 (10.6%)	0.865
Multivariable model (AD: −/+)	52 (65.0%)/28 (35.0%)	28 (14.1%)/171 (85.9%)	<0.001

AD, Alzheimer’s disease; Med, median; M, male; F, Female, MMSE, Mini-Mental State Examination; 20 RU/mL: manufacturer’s instructions.

**Figure 1:**
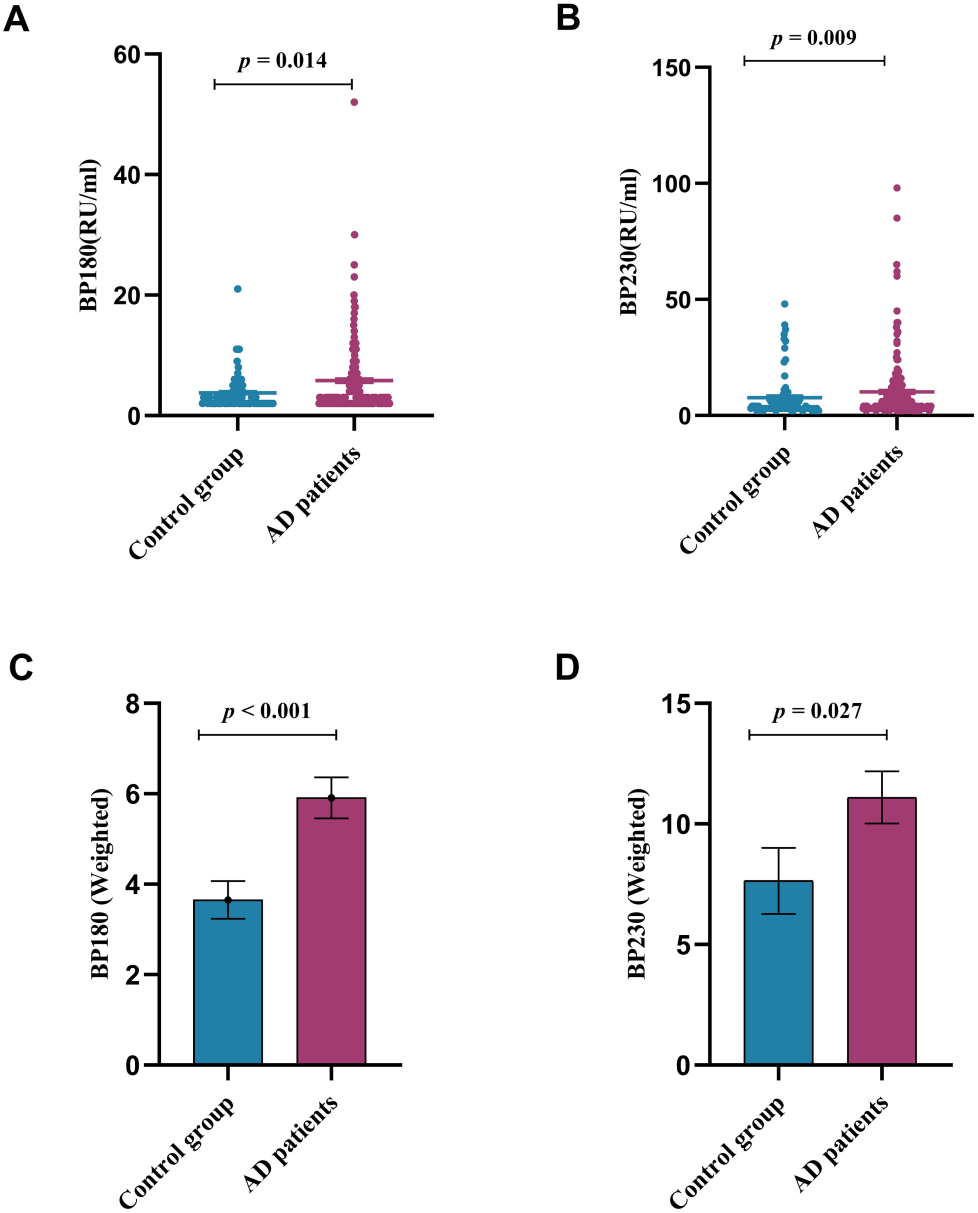
Serum levels and distribution of BP180 and BP230 antibodies in AD patients and controls. **(A)** Serum BP180 levels between AD patients and controls before adjustment (Mann–Whitney test, *p* = 0.014). **(B)** Serum BP230 levels between AD patients and controls before adjustment (Mann–Whitney test, *p* = 0.009). **(C)** Serum BP180 levels in AD patients and controls after inverse probability weighting (IPW) adjustment for age and sex (*p* < 0.001). **(D)** Serum BP230 levels in AD patients and controls after IPW adjustment for age and sex (*p* = 0.027).

Serum levels of BP180 and BP230 antibodies exhibited non-normal distributions in both groups. As shown in Figures 1A,B, anti-BP180 and anti-BP230 antibody levels were significantly higher in AD patients than in controls (BP180: *p* = 0.014; BP230: *p* = 0.009). To minimize potential confounding effects of age and sex, CBPS weighting was applied, resulting in excellent covariate balance between groups (all standardized mean differences <0.1; age and sex SMDs reduced from >0.7 to 0.000). The effective sample size (ESS) after weighting was reduced, indicating appropriate weight distribution and the formation of a balanced pseudo-population.

After weighting, regression analyses confirmed that both biomarkers remained significantly elevated in AD patients. Specifically, BP180 levels were higher in AD patients compared with controls (*β* = 2.257, 95% CI: 1.102–3.412, *p* < 0.001; Figure 1C), as were BP230 levels (*β* = 3.432, 95% CI: 0.395–6.468, *p* = 0.027; Figure 1D). These findings indicate a robust and statistically significant increase in circulating BP180 and BP230 autoantibodies in AD patients after adjustment for potential confounders.

Multivariable logistic regression analysis identified BP180 (OR = 1.149, 95% CI: 1.056–1.275, *p* = 0.004), age (OR = 1.176, 95% CI: 1.110–1.254, *p* < 0.001), and female sex (OR = 5.318, 95% CI: 2.853–10.272, *p* < 0.001) as independent predictors of AD (Table 2). Using the manufacturer-recommended positivity threshold of 20 RU/mL, BP180 autoantibodies were detected in 7 of 199 patients with AD (3.5%) and in 1 of 80 control subjects (1.3%). BP230 autoantibodies were observed in 21 of 199 AD patients (10.6%) and in 9 of 80 controls (11.3%). When the Multivariable Model was applied, it identified 28 (35.0%) individuals as AD-positive in the control group, while in the AD group, it correctly identified 171 (85.9%) true AD patients, with 28 patients (accounting for 14.1%) were classified as negative by the model. Although BP230 showed a more significant difference in univariate analysis, this difference did not retain statistical significance in the multivariable model, and thus BP230 was not included in the final multivariable model.

**Table 2:** Independent predictors in the multivariable model (BP180 + age+sex) for AD patients.

	Multivariable model	
	OR, 95% CI	*p-*value
Age, years	1.176 (1.110–1.254)	<0.001
Sex (female vs. male)	5.318 (2.853–10.272)	<0.001
BP180 (RU/mL)	1.149 (1.056–1.275)	0.004

OR, odds ratio; 95% CI, 95% Confidence Interval.

**Figure 2:**
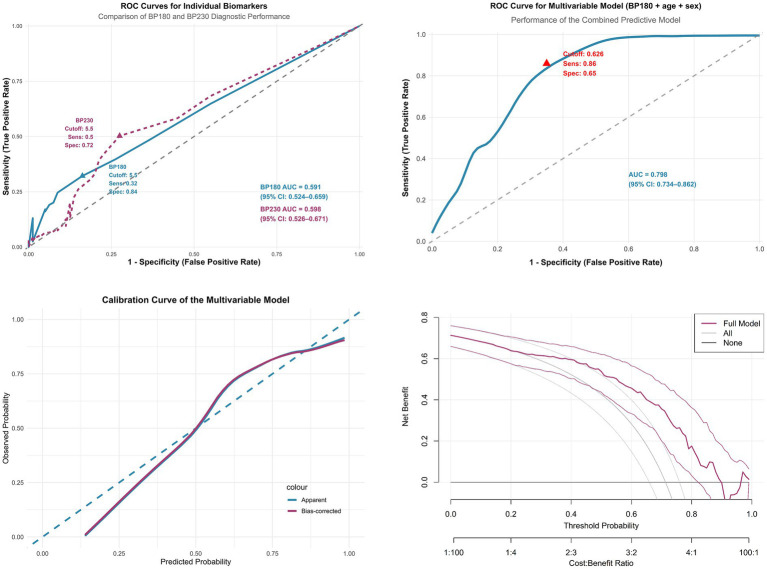
Diagnostic and predictive performance of serum BP180 and BP230 for AD, and evaluation of a multivariable model incorporating BP180, age, and sex. **(A)** Receiver operating characteristic (ROC) curves of BP180 and BP230 in diagnosing AD. **(B)** ROC curve of the multivariable prediction model combining BP180, age, and sex. **(C)** Calibration curve of the multivariable model demonstrating good agreement between predicted and observed AD risk. **(D)** Decision curve analysis (DCA) illustrating the net clinical benefit of the multivariable model across a range of threshold probabilities.

A diagnostic model incorporating these independent predictors demonstrated good discriminative performance, with an AUC of 0.798 (95% CI: 0.734–0.862). At the optimal cutoff value (predicted probability = 0.626), the model achieved a sensitivity of 85.9%, a specificity of 65.0%, and an overall accuracy of 79.9% (Figure 2B). Sensitivity represents the proportion of AD patients correctly classified as positive by the model at the optimal cutoff. Compared with individual biomarkers alone (BP180: AUC = 0.591; BP230: AUC = 0.598) (Figure 2A), the multivariable model integrating age and sex substantially improved the discrimination between AD patients and controls. Calibration analysis further demonstrated good agreement between predicted and observed probabilities, with the bias-corrected curve closely approximating the ideal reference line across the range of risk (Figure 2C), indicating adequate model calibration. In addition, decision curve analysis (Figure 2D) showed that the multivariable model provided a higher net benefit than both the treat all and treat none strategies across a wide range of threshold probabilities, supporting its potential clinical utility. Overall, these results indicate that integrating clinical variables with serological biomarkers substantially enhances both the discriminative performance and clinical applicability of the model, outperforming the use of any single biomarker alone.

**Figure 3:**
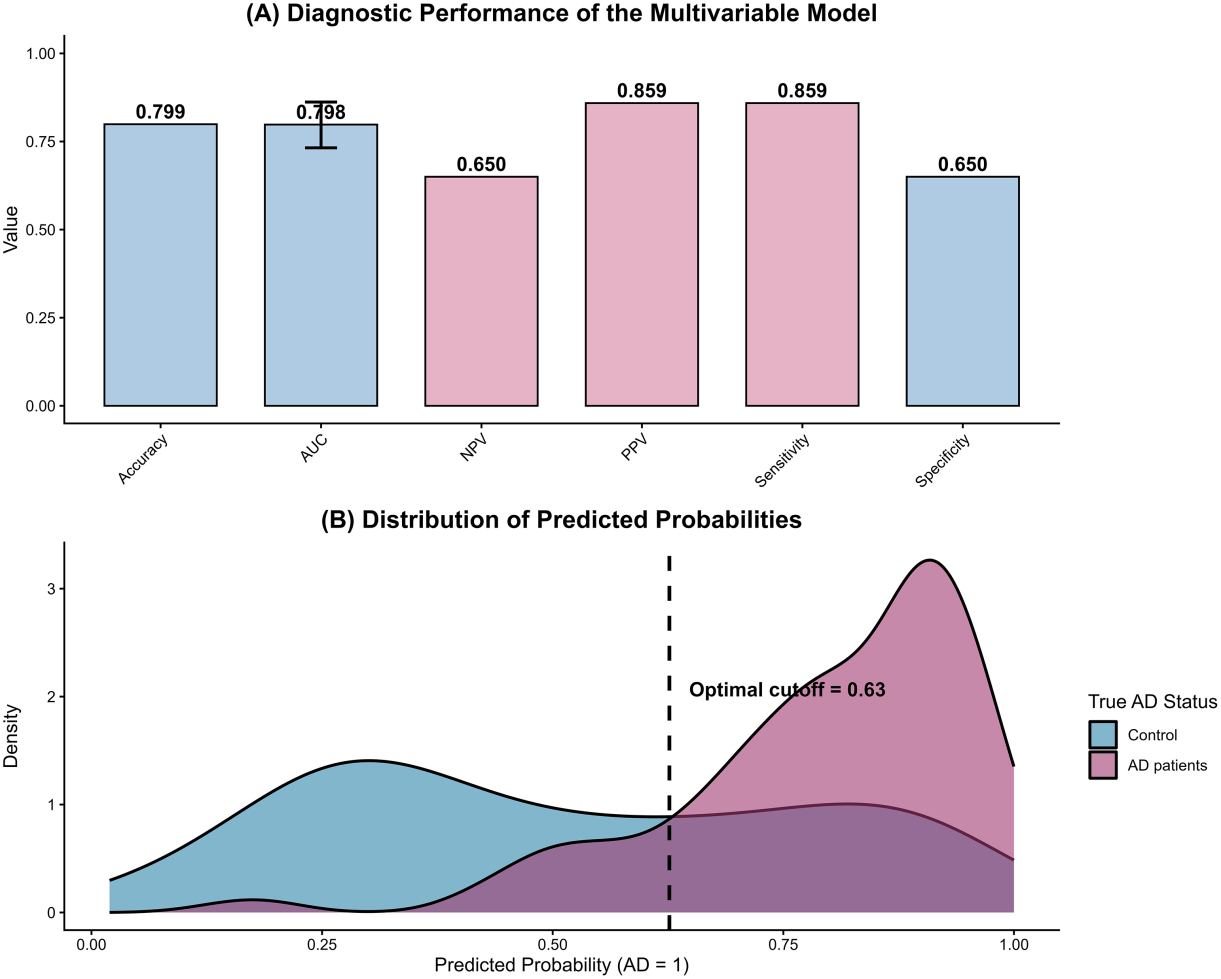
Predicted probability distribution and optimal cutoff of a multivariable model (BP180 + age + sex) for AD patients. **(A)** Diagnostic performance metrics of the multivariable model (incorporating BP180, age, and sex). Metrics include: *Accuracy*: Proportion of all predictions that are correct; *AUC (*a*rea under the receiver operating characteristic curve):* Measures the model’s ability to distinguish between AD patients and controls; *NPV (*n*egative predictive value)*: Proportion of true negatives among all predicted negatives; *PPV (*p*ositive predictive value)*: Proportion of true positives among all predicted positives; *Sensitivity (*r*ecall)*: Proportion of true AD patients correctly identified; *Specificity*: Proportion of true controls correctly identified. **(B)** Distribution of predicted probabilities for AD (AD = 1) stratified by true AD status (control: blue; AD patients: red). The dashed line indicates the optimal cutoff (0.63) for classifying individuals as “AD” or “Control,” determined to maximize clinical utility.

Figure 3 summarizes the diagnostic performance of the multivariable prediction model incorporating BP180, age, and sex. As shown in Figure 3A, the model demonstrated good overall performance, with an accuracy of 0.799 and an AUC of 0.798, indicating reliable discriminative ability between AD patients and controls. In addition, clinically relevant performance indices further supported its utility, including a sensitivity of 0.859, specificity of 0.650, positive predictive value (PPV) of 0.859, and negative predictive value (NPV) of 0.650, suggesting a balanced capacity for both identifying true AD cases and correctly excluding non-AD individuals. As illustrated in Figure 3B, the distribution of predicted probabilities showed a clear separation between AD patients and controls, with AD cases generally exhibiting higher predicted risk scores and controls clustering toward lower probabilities. The vertical dashed line indicates the optimal classification cutoff (predicted probability = 0.630), which was determined to maximize clinical utility. This threshold provided effective stratification of individuals into AD and Control groups, with limited overlap between distributions, further supporting the robustness and potential clinical applicability of the model.

Sex-stratified analyses revealed significant differences in model performance between male and female participants. In males, the model demonstrated relatively balanced discriminative ability, with an AUC of 0.827, accuracy of 73.4%, sensitivity of 82.1%, and specificity of 66.7%, indicating a stable classification performance across both positive and negative cases. In contrast, performance in females was characterized by high overall accuracy (85.4%) but marked imbalance between sensitivity and specificity. Although specificity remained high (96.9%), sensitivity was substantially reduced (25.0%), resulting in an AUC of 0.638. This pattern suggests that the model tended to correctly exclude non-AD female individuals while failing to identify a considerable proportion of true AD cases. Among true-positive cases, 71.9% were female; however, this finding likely reflects the higher proportion of females in the study cohort rather than a sex-specific enhancement in model performance. The observed sex-related differences may be attributed to several factors, including baseline sample imbalance, differences in antibody distribution patterns between sexes, and potential biological heterogeneity in immune responses. Collectively, these findings indicate that while the model performs robustly in males, its sensitivity in females is limited, highlighting the importance of considering sex-specific effects in the development and application of serology-based diagnostic models.

Spearman correlation analysis was performed to assess the relationships between serum BP180 and BP230 levels and cognitive function, as measured by MMSE scores, in AD patients. BP180 levels were significantly and negatively correlated with MMSE scores (*r* = −0.354, *p* < 0.001), and BP230 levels also showed a significant negative correlation with MMSE scores (*r* = −0.211, *p* = 0.003), indicating that higher antibody levels are associated with lower cognitive performance.

## Discussion

Serum BP180 and BP230 autoantibody levels were higher in AD patients than in controls; however, using the manufacturer-defined cutoff (20 RU/mL), only a small proportion of patients were classified as positive, indicating limited diagnostic sensitivity. A multivariable model incorporating BP180, age, and sex achieved improved discriminative performance (AUC = 0.798) and correctly identified 85.9% of AD cases. These findings suggest that integrating serological and demographic variables improves diagnostic performance by capturing the heterogeneous immune profile of AD.

Although BP230 showed a more significant difference in univariate analysis, this difference did not retain statistical significance in the multivariable model, these findings suggest that BP230 contributes modestly to the serological immune profile of AD and may represent a weaker but detectable component of disease-associated immune alterations, rather than a dominant signal. Previous studies (Schmidt et al., 2014) have reported that BP230 autoantibodies can be detected in elderly individuals with pruritic or preclinical forms of bullous pemphigoid, potentially representing an early or nonspecific autoimmune response in aging populations. In addition, rare case reports (Zheng et al., 2018) have described the coexistence of BP230-type bullous pemphigoid and dementia; however, causal inference remains limited due to small sample sizes. Importantly, in the present larger cohort, BP230 not only showed a significant association with AD but was also correlated with reduced MMSE scores, suggesting a potential link with cognitive decline. This finding differs from previous smaller studies (Kokkonen et al., 2017) and may indicate that BP230-associated immune responses are more readily detectable in larger samples and may carry clinical relevance beyond a purely incidental phenomenon.

Substantial variability has been reported in BP180 and BP230 autoantibody positivity rates among patients with AD, with BP180 positivity ranging from 5.8 to 47.9% across previous studies (Kokkonen et al., 2017; Wang et al., 2021; Foureur et al., 2006), which may partly explain the relatively low positivity observed in our cohort under the manufacturer-recommended cutoff. This variability likely reflects differences in assay methods, patient populations, and diagnostic thresholds. In the present study, we applied the manufacturer-recommended cutoff value (20 RU/mL), which may have reduced sensitivity in AD cohorts. These observations suggest that currently established cutoffs may not be directly transferable to neurologically associated conditions such as AD. Future multicenter studies are warranted to establish disease-specific reference ranges, and collaborative efforts between manufacturers and clinical centers may help develop optimized, AD-tailored thresholds to improve diagnostic utility.

Consistent with previous studies (Kokkonen et al., 2017; Wang et al., 2021), BP180 showed a stronger association with cognitive decline in AD than BP230, as reflected by its higher unstandardized regression coefficient. After adjusting for age and sex, serum BP180 levels were significantly higher in AD patients than in controls (*p* < 0.001), and showed a stronger association with MMSE scores (*p* < 0.001) compared with BP230 (*p* = 0.003). Kokkonen et al. (2017) reported no significant difference in BP230 levels between groups (*p* = 0.058), although a trend toward higher levels in AD was observed.

In the study by Kokkonen et al. (2017), cerebrospinal fluid biomarkers (Aβ1–42, Tau, and p-Tau181) showed significant differences between AD patients and controls (*p* < 0.001), whereas no correlation was observed between these biomarkers and serum antibody levels. Wang et al. (2021) reported that sera from AD patients reacted with a 180-kDa brain protein, full-length BP180, and its NC16A fragment; however, no BP-like skin lesions were observed during a 3-year follow-up, suggesting that anti-BP180 autoantibodies are not associated with short-term BP onset. Similarly, Brick et al. (2014) found that BP patients had an increased risk of developing PD (HR 8.56; 95% CI 1.55–47.25; *p* = 0.010) and other neurological disorders (HR 2.02; 95% CI 1.17–3.49; *p* = 0.010) compared with non-BP individuals.

Nieto-Benito and Suárez-Fernández (2025) analyzed 257 BP patients and found that 102 had comorbid neurological disorders, with AD being the most frequent. These findings suggest a close association between BP and neurological diseases, whereas AD patients with elevated BP180 antibodies do not appear to develop BP-related cutaneous manifestations. In their cohort, most anti-BP180-positive AD patients were male (14/23, 60.9%) and older than controls (74.0 vs. 72.2 years; *p* < 0.050). In our cohort, among 171 correctly identified AD patients (true positives), 48 (28.1%) were male and 123 (71.9%) were female. This distribution likely reflects the sex composition of the study population rather than sex-specific differences in diagnostic performance. Overall, these observations indicate that sex-related differences in antibody positivity patterns may exist across studies; however, their impact on diagnostic accuracy remains uncertain. Further sex-stratified analyses in larger independent cohorts are needed to clarify whether sex acts as a true modifier of model performance.

Tuusa et al. (2019) demonstrated through systematic epitope mapping that IgG autoantibodies in patients with MS and AD predominantly target intracellular and mid-extracellular regions of BP180 (e.g., FP3 and FP4), rather than the immunodominant NC16A domain (FP5) implicated in bullous pemphigoid. This epitope specificity may explain why these antibodies detect full-length BP180 in immunoblotting but show limited binding to native cutaneous basement membrane structures in ELISA or indirect immunofluorescence assays. Consistently, Wang et al. (2021) observed no BP-like skin manifestations during a 3-year follow-up of BP180-positive AD patients. Similarly, Messingham et al. (2016) reported that autoantibodies from Parkinson’s disease (PD) patients colocalize with tyrosine hydroxylase-positive neurons but do not react with skin tissue, suggesting a neuronal rather than cutaneous antigenic source.

Collectively, these findings suggest that BP180 autoantibodies in neurological disorders preferentially recognize cryptic epitopes that may reflect neuronal injury rather than pathogenic skin-directed autoimmunity. This may explain the absence of cutaneous manifestations despite seropositivity in AD patients.

This study has several limitations. First, it was based on a single-center retrospective cohort with a limited sample size. Although inverse probability weighting was used to reduce baseline imbalance, potential selection bias cannot be fully excluded, and validation in prospective multicenter cohorts is needed. Second, the model included only age, sex, and serum autoantibodies, without accounting for other established AD risk factors such as genetics and comorbidities, which may limit its predictive completeness. We also observed a sex-related difference in model performance (AUC = 0.827 in males vs. 0.638 in females), but the underlying mechanism remains unclear and requires further investigation. Finally, the lack of external validation and absence of comparison with established biomarkers such as Aβ and p-tau further limit clinical generalizability.

## Conclusion

The multivariable model incorporating BP180, age, and sex demonstrated good discriminative performance in distinguishing AD patients from controls and outperformed individual biomarkers. Integrating clinical and serological markers may improve the diagnostic performance for AD.

## Data Availability

The original contributions presented in the study are publicly available. This data can be found here: https://doi.org/10.6084/m9.figshare.32509413.
